# Promoting genetic and genomic practices among allied healthcare professionals and nurses: a systematic review

**DOI:** 10.1038/s41431-026-02038-5

**Published:** 2026-02-27

**Authors:** Tharushini Anandam, Sanne Peters, Mariana Lauretta, Angela Morgan, Stephanie Best

**Affiliations:** 1 Melbourne School of Health Sciences, Faculty of Medicine, Dentistry and Health Sciences, The University of Melbourne, Melbourne, Australia; 2 Translational Centre for Speech Disorders, Murdoch Children’s Research Institute, Melbourne, Australia

**Keywords:** Psychology, Human behaviour

## Abstract

Genetic practices are increasingly recognised as essential components of modern healthcare. Allied health professionals and nurses are ideally placed to initiate discussions about genetic investigations with patients and families. However, there are known barriers such as a lack of confidence and knowledge. A key step to addressing these barriers is identifying implementation strategies that support the integration of genetics across healthcare disciplines. We conducted a systematic review to identify empirical and conceptual implementation strategies to support genetic practices among allied health professionals and nurses. We searched CINAHL, Embase, Emcare, Medline, Scopus, and Web of Science for articles published from 2020. Twenty-eight full-text articles were included in the review. Identified implementation strategies were mapped to the Theoretical Domains Framework (TDF) to identify key areas for behaviour change. Empirical strategies, including workshops, online learning, case-based education, and leadership development, demonstrated positive effects on supporting genetic integration into clinical practice. Conceptual strategies identified included: (1) education/learning, (2) professional development, (3) policy, (4) evaluation tools, and (5) educational resources. The TDF domains of *Knowledge, Social Influences*, and *Social/Professional Role and Identity* were commonly found, while the TDF domains of *Intentions, Reinforcement, Optimism, Emotion* and *Goals* were underrepresented. Findings demonstrate that empirical and conceptual implementation strategies lack evaluation and tend to focus solely on commonly targeted domains. Future research is needed to investigate the feasibility and effectiveness of implementation strategies, explore the underrepresented domains, and support efforts to increase genetic literacy and practices among allied health professionals and nurses.

## Introduction

Genetic and genomic clinical practices have increasingly become recognised as essential components in various healthcare settings [1]. Testing based on next-generation sequencing, including exome and whole genome sequencing [2, 3], is becoming more common due to reduced costs, more efficient return of results, and greater accessibility [4, 5]. Resultant genetic data have revealed improved diagnosis, prognostic counselling and more targeted therapies. These advances in the genetic era have increased the demand for traditionally non-genetic health professionals to become more aware of and engaged in genetics and genomics. Genetic professionals (e.g. genetic counsellors and clinical geneticists) are trained to order, interpret and discuss genetic results. However, the shortage of trained clinical geneticists and genetic counsellors leads to longer patient waiting times and higher caseloads [6]. The gradual demands for genetic services have resulted in the mainstreaming of genetics into the roles of traditionally non-genetic healthcare professionals [7]. For example, nurses are increasingly responsible for identifying at-risk individuals and initiating genetic discussions with patients, coordinating genetic testing, or delivering genetic test results [8].

Although not all allied health and nursing professionals are directly responsible for initiating genetic testing referrals, they can play an important role in supporting patients by assisting them in understanding their genetic diagnoses and facilitating access to appropriate services when needed. Previous research, among 3600 American allied health professionals, reported 70% had discussed the genetic basis of health concerns with their clients [9]. Given the circumstances, there is a need for improving genetic literacy among health care professionals to support the integration of genetic practices.

Further to being at the forefront of patient care and potentially being the first to identify a possible need for genetic testing [10], it is now commonplace for allied health professionals to receive referrals for clinical management of children who have already received a genetic diagnosis. AHPs need adequate genetic knowledge when they are the first to observe signs suggestive of an underlying genetic condition, so they can recognise when a referral is warranted. They also need this knowledge when treating children with an existing genetic diagnosis, as genomic information shapes treatment goals, clinical outcomes, therapy approaches, and the support provided to families. Yet health professionals express a lack of knowledge of why and how they should consider these diagnoses in relation to their usual clinical care. Existing studies have documented the need for genetic literacy among various healthcare professionals, including speech-language pathologists (SLPs) [11], occupational therapists [12], audiologists [13], physiotherapists [14], optometrists [15], and nurses [16]. A common barrier identified among healthcare professionals, including SLPs, audiologists [17], and nurses [18, 19], in engaging with genetics in clinical practice is a lack of genetic knowledge and training. Although healthcare professionals’ express interest in learning more about genetics, they lack access to credible resources such as covering genetics in their training and guidelines tailored to their profession [20, 21]. Despite efforts by various healthcare organisations to expand genetic education, significant gaps in education, training, and resources persist, posing challenges to the awareness and engagement of genetics and genomic practices [22, 23].

Further barriers to bringing genetics into clinical discussions with families and patients have been reported. Lauretta et al. identified several other barriers among SLPs, along a range of touchpoints with families, before, during, and after the genetic testing process [24]. Barriers at these three points included uncertainty about professional role responsibilities in discussing genetics, lack of experience in the genetic field, and concerns about the potential emotional impact of genetic diagnoses, making healthcare professionals hesitant to discuss either genetic referrals or even genetic results and what they may mean for the clinical care of their patients. A lack of structured support systems, such as access to other healthcare professionals and experts, has also been identified as a barrier towards the promotion of genetics into routine practice [7, 25, 26]. These barriers, ranging from uncertainty about professional roles to limited experience and lack of structured support systems suggests that many healthcare professionals currently lack the necessary resources and collaborative networks to effectively engage with genetic information.

While previous research has highlighted knowledge as a prominent barrier to incorporating genetics into practice among health care professionals, it is necessary to acknowledge that behaviour change includes multiple factors. The Theoretical Domains Framework (TDF) is a comprehensive framework used to identify and address myriad factors influencing behaviour [27]. Barriers and enablers to a behaviour can be mapped onto the 14 TDF domains, which are grouped into cognitive, social, environmental, and affective determinants of behaviour change [28]. TDF domains provide insights into barriers influencing behaviour, beyond the usual suspects of education, enabling the development of targeted implementation strategies that increase the likelihood of the behaviour change.

This review aimed to examine the experiences of healthcare professionals, specifically nurses and allied health professionals such as speech pathologists, occupational therapists, audiologists, physiotherapists, and optometrists, to capture a broad spectrum of practitioners who have not traditionally worked in genetics. Hence, the review focused on empirical and conceptual implementation strategies that support the integration of genetic practices among these healthcare professionals. Empirical strategies refer to evidence-based interventions that have been tested or evaluated in practice, while conceptual strategies refer to proposed approaches that have not been tested. Moreover, mapping strategies to the TDF allowed us to identify which behavioural domains were targeted to support the integration of genetic practices among nurses and allied health professionals. This process also permited identification of domains that are currently well represented in the literature and which remain underrepresented.

### Objectives

The objectives of this systematic review were to:

1. To review empirical and conceptual strategies that support genetic practices among allied healthcare professionals and nurses.
2. To map the identified empirical and conceptual strategies onto the TDF.

## Methods

A systematic review approach was selected to enable a rigorous and comprehensive synthesis of strategies used to support genetic and genomic practice [29]. Systematic reviews are appropriate when addressing specific questions about intervention effectiveness or practice-related outcomes. The systematic literature review was reported based on the Preferred Reporting Items for Systematic reviews and Meta-Analyses (PRISMA) statement [30]. The study was registered with OSF Registries on December 17, 2024 (10.17605/OSF.IO/WYHA2).

### Information sources

Following advice from a specialist health librarian, we searched six electronic databases: CINAHL, Embase, Emcare, Medline, Scopus, and Web of Science. The objectives were broken down into key terms. To test the search strategy, retrieved results were compared against a set of pre-identified exemplar studies within the scope of the review. The search strategy was refined accordingly, and included, genomic* OR genetic* AND referral or counsel* OR test* OR consult* OR practice* OR knowledge OR training OR education. The final, full search strategy can be accessed in Supplementary File 1.

### Inclusion and exclusion criteria

Before screening commenced, eligibility criteria were developed through an iterative process of article review and discussions with the research team (see Table 1). The review included studies that focused on empirical and conceptual strategies to support genetic/genomic practices or interventions. Empirical strategies refer to tested practices, such as pre-existing education programs, policies or guidelines. Conceptual strategies refer to non-tested suggestions, which could involve fostering collaborations between healthcare professionals or developing of training session. The review included studies with participants from the following professions: nurses, allied health professionals (including but not limited to speech pathologists, occupational therapists, audiologists, physiotherapists, and optometrists). To ensure the focus was maintained on these professions, in studies with mixed populations, at least 75% of participants had to be from the target professions. Articles were required to be peer-reviewed and could be quantitative, mixed-method, qualitative, observational, or case studies.

**Table 1:** Eligibility/inclusion criteria.

Inclusion criteria	Exclusion criteria
Participants must be either speech pathologists, nurses, allied healthcare professionals, occupational therapists, audiologists, physiotherapists, or optometrists. The target population is more than 75% in a mixed population.	Participants are not speech pathologists, nurses, allied healthcare professionals, occupational therapists, audiologists, physiotherapists, or optometrists. The target population is less than 75% in a mixed population.
Studies that include existing strategies to support genetic/genomic practices/interventions	Studies that don’t include existing strategies to support genetic/genomic practices/interventions
Studies that identify proposed strategies to support genetic/genomic practices	Studies that do not identify proposed strategies to support genetic/genomic practices

Studies were excluded if participants were not from: speech pathology, nursing, occupational therapy, audiology, physiotherapy, or optometry. The review also excluded strategies targeting medical clinicians. Additionally, studies with mixed populations where less than 75% of participants were from these professions, as well as reviews, chapters, and commentaries, were excluded. The final database search was conducted in February 2024. Searches were limited to English-language publications from 2020 onwards. This date restriction was chosen to ensure the review reflected the timing of integration of NGS in routine clinical practice [5].

### Selection process

After the search was conducted, all identified articles were collated and uploaded into EndNote 21(Clarivate Analytics, USA) to manage the references and remove duplicates. The EndNote file was then imported into Covidence (Veritas Health Innovation, Australia) for screening of studies, extraction of data and removal of duplicates. Titles and abstracts were screened by two independent reviewers (TA, ML) to assess their eligibility based on the inclusion criteria. The full texts of the eligible articles were retrieved and screened by two independent reviewers (TA & SB or SP) against the inclusion criteria. Reviewers (TA & SB or SP) reached consensus on 40 of 48 articles, resulting in an overall percent agreement of 83.3%. Any disagreements between reviewers at each stage of the selection process were resolved through discussion or with the inclusion of a third reviewer (SB or SP), at regular project meetings.

### Data extraction and analysis

**Fig. 1:**
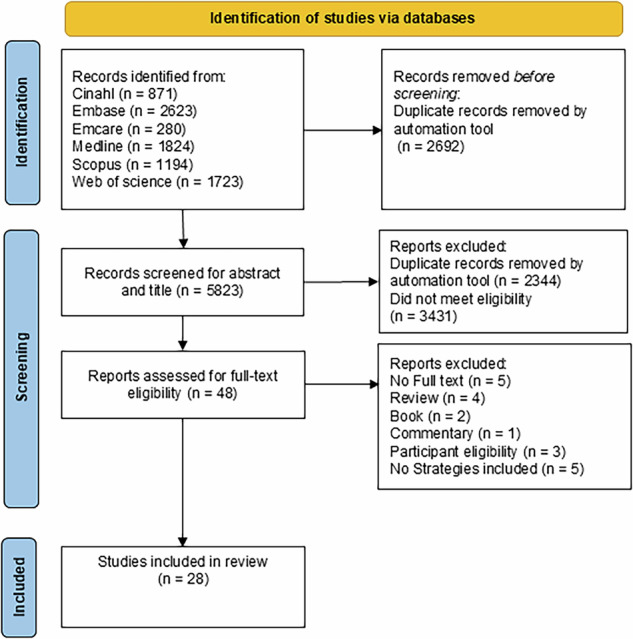
PRISMA flow diagram.

A PRISMA flow diagram was generated to present the results of the search [30](see Fig. 1). Data were extracted from articles included in the review to respond to the research objectives (see Supplementary File 2). The final data extraction table consisted of the following information: author, year, article type, country of origin, population, sample size, key theme related to genetic practices, strategy tested (e.g. existing/tested) or proposed/not tested), measures (e.g. surveys, interviews), study outcome and whether a theoretical framework was applied. One extractor (TA) extracted the data from all included studies. To ensure quality, a random sample of extractions was cross-checked for accuracy and consistency by a second extractor (SB). Any disagreements were resolved through discussion at regular project meetings. Once data extraction was completed, strategies were inductively coded into strategy keywords that captured their core behavioural focus (TA, SB). Coding strategies into keywords enabled a consistent approach to mapping them onto the TDF.

For empirical strategies, all studies associated with the same strategy keyword were collated, noting that studies associated with more than one strategy keyword. Each strategy keyword was then mapped onto the most relevant TDF domain(s) (TA, SB) [28, 31]. This mapping process allowed us to quantify the frequency of the represented TDF domains within empirical strategies. The same process was applied to conceptual strategies, enabling comparison between empirical and conceptual approaches. Mapping decisions were reviewed collaboratively (TA, SB), and any discrepancies were resolved through discussion to ensure consistency. A heat map was then generated to visually depict the frequency of the TDF domain that was targeted across the included studies.

### Article quality appraisal

All articles included in the review were independently assessed (TA) using the Mixed Methods Appraisal Tool (MMAT), and a second reviewer (SB) independently performed quality assessment of a subset to ensure reliability [32]. MMAT evaluated the methodology of each study based on the nature of the study designs, including categories such as randomised controlled trials, non-randomised studies, qualitative studies, and mixed-methods studies. For each included study, the appropriate category was chosen and appraised using the ratings (e.g. yes, no, or unclear).

## Results

A total of 3479 unique articles were retrieved from the database search. Forty-eight articles were assessed against the full-text eligibility. Twenty-eight articles were included in the review after excluding articles based on the inclusion and exclusion criteria. The PRISMA diagram presents the number of studies retrieved (Fig. 1).

Study characteristics of the included articles are summarised in Table 2. While most were conducted in the USA (*n* = 6) [33–38], followed by Canada (*n* = 4) [39–42], Australia (*n* = 2) [24, 43], Turkey (*n* = 2) [44, 45], and China (*n* = 2) [46, 47]. The remaining studies were conducted across several other countries. The majority of the studies (*n* = 18) [33, 35–37, 41, 42, 44–54] used a quantitative design, using surveys or questionnaires, with a few mixed methods studies (*n* = 3) [43, 55, 56], qualitative studies (*n* = 5) [24, 39, 40, 57, 58] and descriptive studies (*n* = 2) [38, 59]. Most studies included participants with a nursing background (*n* = 24), while the remaining studies included occupational therapists [57], optometrists [48], speech pathologists [24], and the collective term allied health professionals [54]. Most studies utilised specific frameworks to support the integration of genetic strategies, including: Canadian Nursing and Genomics Framework (*n* = 3) [39, 40, 42]; Rogers’ Diffusion of Innovation Theory (*n* = 2) [46, 47]; while Lewin’s Action Research Principles [43]. The Consolidated Framework for Implementation Research [44], Theoretical Domains Framework (TDF) [24], R.I.S.E. 2 Genomics Framework [58] and Johnson and Aragon’s (2003) Framework [39] were each referenced in one study.

**Table 2:** Summary of included studies.

Author/publication year	Country	Population/ sample size	Strategy Reported- keywords	Methods/Measures	Tested (yes/no)	Results/Outcome relevant to genomic Strategies
Alexander et al. [43]	Australia	226 nurses and 8 Midwives	Education workshop Educational content Continuing education Role of senior staff Educational/online resources Online learning	Mixed methods Survey Feedback and evaluation form	Yes	The workshop series showed significant improvements in participants’ confidence. Participants expressed a strong desire for more education on topics like genetic testing interpretation, with learning needs increased from 52% to 89% post-workshop. Many participants expressed an interest in applying what they learned to their practice and even pursuing further training or careers in genetics.
Britten-Jones et al. [48]	Australia and New Zealand	516 optometrists	Workshops and continuing education Educational content Collaboration across interdisciplinary	Quantitative Survey	No	–
Carlsson and Limoges [39]	Canada	Nurses across Canada from various healthcare setting	Curriculum development Undergraduate/postgraduate education Collaboration with genomic experts Collaborating with genomic organizations	Qualitative -	No	–
Carpenter-Clawson et al. [55]	United Kingdom	153 cohort nurses	Postgraduate education Synchronous learning (useful point to note) Educational content Continuing education Educational/online resources Online learning	Mixed methods Survey	yes	The course informed the development of a new Postgraduate Certificate in Genomics, which expands the module to include additional training in communication, genetic counselling, and setting up nurse-led genomic services.
Ceylan et al. [44]	Turkey	121 registered nurses	Training program Reminders Educational content Continuing education Educational/online resources Online learning Web-based education	Quantitative Genetics and Genomics in Nursing Practice Survey (GGNPS)	yes	Over 70% of nurses reported that genetics was not included in their education, and over 90% had not attended a genetics-focused course. After training, knowledge scores and attitudes toward genetics improved significantly. Confidence and perceived social support for genetic practices increased, and recognition of the importance of genetics in common diseases rose from 24.8% to 76.9%. However, actual application in clinical decisions showed only modest improvement (5.8% to 7.5%).
Chiu et al. [40]	Canada	Nurse leaders from educational institutions, regulatory bodies, professional associations, and specialty practice groups.	Raising awareness and fostering Positive attitudes Collaborative interdisciplinary Advocacy for financial investment Curriculum development Development of a national genomic strategy Development of a genomic literacy toolkit	Qualitative The Assessment of Strategic Integration of Genomics Across Nursing (ASIGN) Maturity Matrix	No	–
Chow et al. [49]	Hongkong	234 nursing students	Educational resources Continuing education Undergraduate/postgraduate education Professional development Comprehensive assessment of genetic/genomic Literacy Develop courses International collaboration	Quantitative Genetics and Genomics Nursing Practice Survey (GGNPS) Genomic Nursing Concept Inventory (GNCI)	No	–
Dagan et al. [50]	Israeli	423 nurses	Education program Role of experts/ senior staff Change of policies Interdisciplinary collaboration Continue education	Quantitative Questionnaire (genomic knowledge, self-epistemic authority (SEA), the perceived importance of genomics, and the performance of genomic skills)	No	–
Dewell et al. [41]	Canada	undergraduate nursing students (*n* = 207) and faculty (*n* = 13) in a school of nursing	Develop Evidence-Based Educational Intervention Examine accreditation standards Develop courses Influence of nurse educators	Quantitative Genomic Nursing Concept Inventory (GNCI)	No	–
Dumo et al. [51]	Finland and Philippines	250 undergrad nursing students	Development of a web-based education Education resources Content structure for flexible learning Undergraduate education	Quantitative Genomic Nursing Concept Inventory	No	–
Henly et al. [57]	USA	20 occupational therapists	Training program Professional development opportunities Interdisciplinary collaboration Education resources	Qualitative Semi-structured interviews	No	–
Kawasaki et al. [52]	Japan	23 public health nurses	Training program Case studies Educational content Continuing education Educational/online resources	Quantitative Questionnaire (cognitive, affective, psychomotor)	Yes	The mean total score across all domains increased significantly from 19.3 to 28.5 after training. Cognitive, affective, and psychomotor domains each showed marked improvement. Notably, 10 of 23 participants felt able to address genetics-related concerns post-training, compared to none before. While younger nurses initially scored higher, age-related differences disappeared after training. Qualitative feedback highlighted greater awareness of roles in genetic education and referrals, with key themes including providing accurate information and referring to specialized services.
Kronk et al. [33]	United States	132 Nurses with a doctoral degree and doctoral nursing students	Training program Postgraduate education Asynchronous Educational content Educational/online resources Professional development Online learning	Quantitative Genomic Nursing Concept Inventory and Essential Competencies in Genetics and Genomics for Nurses with Graduate Degrees	yes	GNCI scores improved significantly across all categories, with a mean increase of 5.53 points. Self-reported genomic competency scores also rose notably, from 1.80 to 2.91 across 38 competencies. A positive correlation between knowledge and confidence was observed, confirming the course’s effectiveness. No significant differences were found between doctoral students and degree holders. The “Theory to Application” course proved to be an effective online tool for enhancing genomic understanding, with potential for broader application in health professions.
Kronk et al. [34]	United States	83 doctoral-level nurses	Postgraduate education Educational content Continuing education Educational/online resources Case studies Undergraduate education Develop/review curriculum Online learning	Quantitative Genomic Nursing Concept Inventory	Yes	98% of participants reported enhanced knowledge and satisfaction with the course, and most committed to applying genomic knowledge in their teaching and practice.
Laaksonen et al. [59]	Finland	Public health nurses (PHNs) at Tampere University of Applied Sciences (TAMK)	International collaboration Education program Education Courses (standalone) Training Case studies	Descriptive -	no	–
Lauretta et al. [24]	Australia	12 paediatric speech language pathologists	Training Tailored education Professional Development Interdisciplinary collaboration Clinical Supervision Gain experience/exposure Support from HCP Resources	Qualitative Semi-structured Interview	no	–
Limoges et al. [58]	Canada and Finland	21 graduate students (10 Canadian and 11 Finnish registered nurses)	Postgraduate education Comprehensive assessment of genetic/genomic literacy Leadership collaboration	Qualitative Assessment of Strategies to Integrate Genomics in Nursing, one-to-one interviews, written assignments, and reflections	yes	Participation in the GNL-G project enhanced students’ leadership competencies, even without prior genomics experience. Students developed strategies aligned with key success factors like workforce development and genomics awareness in healthcare. They also deepened their understanding of equity, ethics, and social justice in genomics. The ASIGN tool supported evaluation of practice settings and development of targeted implementation strategies
Limoges et al. [42]	Canada	1012 nurses	Develop curriculum Professional development Interdisciplinary collaboration Establishment of genomic champions Established frameworks for guidance Leveraging nurses’ readiness	Quantitative Genetics and Genomics in Nursing Practice Survey (modified)	No	–
Majstorović et al. [53]	Croatia	53 Croatian undergraduate nursing students	Assessment of genomic literacy Courses Review of curriculum Undergraduate/graduate education International genomic guidelines	Quantitative Genomic Nursing Concept Inventory	No	–
Mathis et al. [35]	United States	23 nursing faculty members from the nursing school	Educational program Educational content Continuing education Educational/online resources Online learning Undergraduate education Develop curriculum	Quantitative Questionnaires	yes	All faculty members reported increased knowledge in genetics and genomics, with 68.2% feeling more confident in developing related curricular content. Over 82% expressed interest in incorporating this content into their courses. Faculty developed 18 curricular threads.
Shields et al. [37]	United States	122 National Association of Neonatal Nurses (NICU)	Continued education Tailored education Develop Curriculum Education resources Education programs	Quantitative Survey	No	–
Smania et al. [36]	United States	88 nursing Faculty members	Workshop - synchronous Educational content Continuing education Educational/online resources Undergraduate education Develop/review curriculum Case studies	Quantitative Genetics and Genomic Nursing Practice Survey (GGNPS) and the Genomic Nursing Concept Inventory (GNCI)	Yes	Faculty confidence in genetics/genomics tasks increased significantly after the workshops. Confidence in referring for genetic counselling rose by 27%, and correct responses on genome sequencing and genetic screening questions increased by 54% and 32%, respectively. Post-workshop, 78% intended to integrate genomics into their courses, and 98% reported enhanced knowledge and satisfaction, with most committing to apply this knowledge in teaching and practice.
Thom and Haw [54]	South Africa	57 allied healthcare professionals	Undergraduate education Educational resources Lectures by genetic counsellors*	Quantitative Questionnaire (assessed awareness, knowledge, and barriers to referring patients for genetic counselling)	No	–
Tonkin et al. [56]	Across the globe (19 countries)	30 nursing experts in health care and genomics	Develop curriculum Continuing education Policy development Development of a national genomic strategy International collaboration Role of senior staff Evaluation tools	Mixed methods Maturity Matrix (ASIGN)	No	–
Wang et al. [46]	China	406 nurses	Tailored Education Training program Social system support Educational efforts on nursing managers and senior Education resources	Quantitative Survey: Genetics and Genomics Nursing Practice Survey (GGNPS) questionnaire	No	–
Walker et al. [38]	United States	nurse practitioners	Undergrads/graduate education Continued education Interdisciplinary collaboration Education resources Digital Platforms”	Descriptive -	no	–
Yeşilçinar et al. [45]	Turkey	385 nurses working in clinical or academic settings	Role of senior staff Post-graduation education Education resources Tailored education	Quantitative Genetics and Genomics in Nursing Practice Survey	No	–
Zhao et al. [47]	China	2118 nurses	Undergraduate education Continue education International collaboration Educational resources Training program Raising awareness among national Nursing Leaders	Quantitative Genetics and Genomics in Nursing Practice Survey	No	–

### Empirical strategies

Various strategies have been employed and tested to support the integration of genetics among allied health professionals, and each strategy is described in relation to the genetic context (Table 3). The empirical strategies include workshops (*n* = 1) [43], web-based education and reminder systems (*n* = 1) [44], leadership development (*n* = 1) [58], and the involvement of senior staff to support genetic integration. The integration of educational and online resources was a common strategy to support genetic literacy, including web-based tools and learning platforms (*n* = 7) [33–36, 43, 44, 52, 55]. Curriculum development and review (*n* = 3) [34–36], undergraduate education programs (*n* = 2) [35, 36], and postgraduate education (*n* = 4) [33, 34, 55, 58] were associated with significant improvements in participants’ genetic knowledge. Finally, case-based learning approaches supported genomic literacy, evaluating both genetic knowledge and its application in practice (*n* = 3) [34, 36, 52].

**Table 3:** Empirical strategies.

Strategy	Reference	Description	Mapped TDF domain(s)
Educational content	Carpenter-Clawson (2023), Alexander (2024), Ceylan (2025), Kronk (2023), Kronk (2024), Kawasaki (2021), Smania (2022), Mathis (2022)	Genetics-related material	*Knowledge, Environmental Context and Resources*
Role of senior staff	Alexander (2024)	Leadership support in promoting genetics integration	*Social/Professional Role and Identity, Social Influences*
Educational/online resources	Carpenter-Clawson (2023), Kronk (2023), Alexander (2024), Ceylan (2025), Kronk (2024), Kawasaki (2021), Smania (2022), Mathis (2022)	Tools (e.g., videos, access to websites) to support learning about genetics	*Environmental Context and Resources*
Online learning	Carpenter-Clawson (2023), Kronk (2023), Alexander (2024), Ceylan (2025), Kronk (2024), Mathis (2022)	Web-based learning	*Knowledge*
Postgraduate education	Kronk (2023), Limoges (2024), Kronk (2024), Carpenter-Clawson (2023)	Genetics integrated in postgraduate education	*Knowledge*
Undergraduate education	Kronk (2023), Smania (2022), Mathis (2022)	Foundational genetics integrated in undergraduate education	*Knowledge*
Synchronous learning/ Asynchronous learning	Carpenter-Clawson (2023)	Real-time genetics learning sessions (e.g., live webinars)/ Self-paced or recordings	*Knowledge, Memory, Attention, and Decision Processes*
Program	Ceylan (2025), Kawasaki (2021), Kronk (2024), Mathis (2022)	Structured program for teaching genetics	*Knowledge, Skills*
Reminders	Ceylan (2025)	Prompts to reinforce the application of genetic knowledge in practice	*Memory, Attention and Decision Processes, Behavioural Regulation*
Web-based education	Ceylan (2025)	Genetic learning and resources are delivered via online platforms	*Environmental Context and Resources, Knowledge*
Case studies	Kawasaki (2021), Smania (2022), Kronk (2023)	Scenarios used to apply genetic knowledge in clinical decision-making	*Knowledge, Skills, Memory, Attention and Decision Processes*
Professional development	Kronk (2024)	Training to enhance genetics competencies	*Beliefs about Capabilities, Social/Professional Role and Identity*
Assessment of genetic literacy	Limoges (2024)	Tools to evaluate understanding of genetic concepts	*Knowledge, Behavioural Regulation*
Leadership	Limoges (2024)	Staff and experts influencing integration of genetics into practice and education	*Skills, Beliefs about Capabilities, Social/professional Role and Identity*
Collaboration	Limoges (2024)	Working across disciplines to support genetics integration	*Social Influences and Social/Professional Role and Identity*
Develop curriculum	Mathis (2022), Smania (2022), Kronk (2023)	Creating or updating education plans to include genetics content	*Knowledge, Environmental Context and Resources*

### Conceptual strategies

Several conceptual strategies were suggested to enhance genomic literacy among healthcare professionals, including: education and training programs (*n* = 9) [24, 36, 46, 47, 50, 51, 57, 59, 60], educational resources (*n* = 9) [24, 37, 38, 45, 47, 49, 54, 57, 60], interdisciplinary collaboration (*n* = 9) [24, 38–40, 42, 48, 50, 57, 60], curriculum development (*n* = 7) [37, 39, 40, 42, 53, 56, 60], continuing education (*n* = 6) [38, 48, 50, 51, 56, 59] (Table 4). The influence of nurse educators, senior staff, and expert support systems (*n* = 7) was identified as a strategy to support integration of genetics [24, 41, 45, 46, 50, 56, 60]. There were also other strategies proposed such as development of standalone or integrated courses (*n* = 6) [41, 48, 49, 51, 53, 59], international collaboration (*n* = 5) [47, 49, 51, 56, 59], tailored education approaches (*n* = 4)[24, 37, 45, 46], professional development opportunities (*n* = 4) [24, 42, 49, 57], development of genomic literacy toolkits and evaluation tools (*n* = 4) [40, 49, 53, 56], undergraduate education (*n* = 4) [38, 47, 53, 54], postgraduate education (*n* = 3) [39, 45, 49], clinical supervision and experiential learning (*n* = 2) [24, 60], use of case studies as practical learning tools (*n* = 2) [51, 59], raising awareness and fostering positive attitudes (*n* = 2) [40, 47], co-designed educational content (*n* = 1) [48], lectures delivered by genetic counsellors (*n* = 1) [54], examination of accreditation standards (*n* = 1) [41]. Digital platforms (*n* = 1) are suggested to support educational delivery [38], and advocacy efforts (*n* = 5), including securing financial investment [40], policy development [50, 56], and alignment with national and international genomic strategies [40, 53, 56] were also identified to support the integration of genetics.

**Table 4:** Conceptual strategies.

Strategy	Reference	TDF domain
Education and training programs	Dumo, 2020; Laaksonen, 2022; Henly, 2023; Wang, 2023; Dagan, 2021; Zhao, 2022; Lauretta, 2024; Lopes-JÃ°nior, 2022; Shields, 2023	*Knowledge, Skills*
Curriculum development	Carlsson, 2022; Chiu, 2024; Limoges, 2024; Lopes-JÃ°nior, 2022; Tonkin, 2020; MajstoroviÄ‡, 2021; Shields, 2023	*Knowledge, Environmental Context and Resources*
Educational resources	Henly, 2023; YeÅŸilÃ§Ä±nar, 2022; Chow, 2023; Thom, 2021; Zhao, 2022; Lauretta, 2024; Lopes-JÃ°nior, 2022; Shields, 2023; Walker, 2023	*Environmental Context and Resources*
Standalone or integrated courses	Britten-Jones, 2024; Chow, 2023; Dewell, 2020; Dumo, 2020; Laaksonen, 2022; MajstoroviÄ‡, 2021	*Knowledge, Skills*
Interdisciplinary collaboration	Britten-Jones, 2024; Carlsson, 2022; Chiu, 2024; Henly, 2023; Lauretta, 2024; Limoges, 2024; Walker, 2023; Dagan, 2021; Lopes-JÃ°nior, 2022	*Social Influences and Social/Professional Role and Identity*
International collaboration	Chow, 2023; Dumo, 2020; Laaksonen, 2022; Tonkin, 2020; Zhao, 2022	*Social Influences and Social/Professional Role and Identity*
Professional development	Henly, 2023; Chow, 2023; Limoges, 2024; Lauretta, 2024	*Social/professional Role and Identity, Belief about Capabilities*
Clinical supervision and experiential learning	Lauretta, 2024; Lopes-JÃ°nior, 2022	*Skills, Social/Professional Role and identity, Belief about Capabilities*
Undergraduate education	Thom, 2021; Zhao, 2022; MajstoroviÄ‡, 2021; Walker, 2023	*Knowledge*
Postgraduate education	Carlsson, 2022; Chow, 2023; YeÅŸilÃ§Ä±nar, 2022	*Knowledge*
Continuing education	Dumo, 2020; Laaksonen, 2022; Dagan, 2021; Tonkin, 2020; Walker, 2023; Britten-Jones, 2024	*Knowledge*
Tailored education approaches	Lauretta, 2024; Shields, 2023; YeÅŸilÃ§Ä±nar, 2022; Wang, 2023	*Knowledge*
Use of digital platforms	Walker, 2023	*Environmental Context and Resources*
Awareness and attitude fostering	Chiu, 2024; Zhao, 2022, Lauretta, 2024	*Belief about Consequences*
Frameworks and leadership readiness	Limoges, 2024	*Social/Professional Role and Identity, Beliefs about Capabilities*
Advocacy and investment	Chiu, 2024; Dagan, 2021; Tonkin, 2020	*Social/Professional Role and Identity, Environmental Context and Resources*
Alignment with genomic strategies	Chiu, 2024; Tonkin, 2020; MajstoroviÄ‡, 2021	*Environmental Context and Resources*
Genomic literacy toolkits and evaluation tools	Chiu, 2024; Chow, 2023; Tonkin, 2020; MajstoroviÄ‡, 2021	*Knowledge, Behavioural regulation*
Support systems and expert influence	Dewell, 2020; Tonkin, 2020; YeÅŸilÃ§Ä±nar, 2022; Dagan, 2021; Wang, 2023; Lauretta, 2024; Lopes-JÃ°nior, 2022	*Social Influences and Social/Professional Role and Identity*
Co-designed content	Britten-Jones, 2024	*Knowledge, Environmental Context and Resources*
Lectures by genetic counsellors	Thom, 2021	*Knowledge, Skills, Social Influences*
Use of case studies	Dumo, 2020; Laaksonen, 2022	*Knowledge, Skills, Memory, Attention and Decision Processes*
Examination of accreditation standards	Dewell, 2020	*Environmental Context and resources*
Targeted education for leadership	Wang, 2023	*Skills, Beliefs about capabilities, Social/ Professional Role and Identity*
Genomic champions	Limoges, 2024	*Social Influences, Social/Professional Role and Identity*
Evidence-based interventions	Dewell, 2020	*Knowledge*

### Theoretical Domains Framework

The empirical and conceptual strategies were mapped to the domains of the TDF, categorising factors that influence the integration of genetics into practice. Both empirical and conceptual strategies reported the use of several resources. For example, within the *Environmental Context and Resources* domain, educational resources were a common strategy used among the empirical strategies [33, 34, 43, 55]. These included online modules, toolkits, and self-directed learning platforms designed to improve accessibility and engagement among health care professionals. *Social Influences* and *Social/Professional Role and Identity* were also frequently targeted domains among the strategies identified in this review. Some studies reported the involvement of senior staff and leadership [43, 50], while others proposed the use of genomic champions and interprofessional training to promote peer influence and support [24, 42]. These strategies recognise the importance of fostering a supportive environment for healthcare professionals.

Across the included empirical strategies, the most frequently mapped TDF domain was *Knowledge* (*n* = 10), highlighting its importance in implementation strategies. *Environmental Context and Resources* (*n* = 4) and *Social/Professional Role and Identity* (*n* = 4) showed moderate representation. *Memory, Attention and Decision Processes* (*n* = 3), *Skills* (*n* = 3), *Social Influences* (*n* = 2), *Behavioural Regulation* (*n* = 2), and *Beliefs about Capabilities* (*n* = 2) were mapped less often (Table 3). While among the identified conceptual strategies, Knowledge (*n* = 12) was again the most frequently, followed by *Social/Professional Role and Identity* (*n* = 9), *Environmental Context and Resources* (*n* = 7), *Skills* (*n* = 6), *Social Influences* (*n* = 5) and *Beliefs about Capabilities* (*n* = 4) were moderately mapped to the conceptual strategies. *Belief about Consequences* (*n* = 1), *Behavioural Regulation* (*n* = 1), and *Memory, Attention and Decision Processes* (*n* = 1) were mapped less often.

**Fig. 2:**
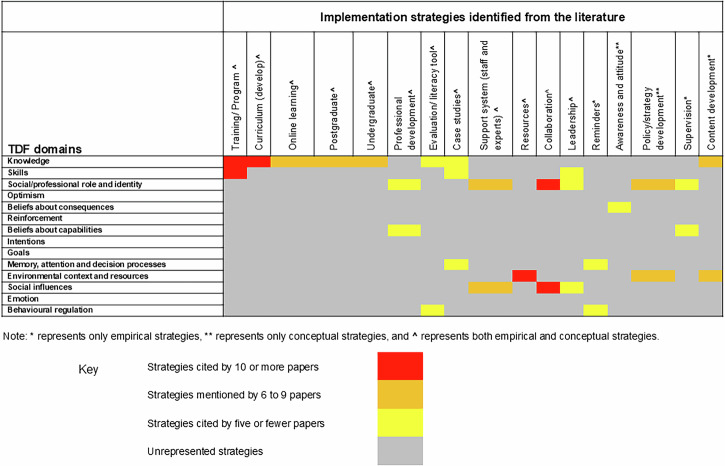
Heat map on implementation strategies and associated TDF domains.

Overall, TDF domains such as *Knowledge, Social Influences*, and *Social/Professional Role and Identity* were the most frequently represented. These domains were associated with various strategies, for example, *Social Influences* was mapped onto support system, collaboration, leadership and supervision, including the involvement of genomic champions to promote engagement with genetic and genomic practices (Fig. 2). *Intentions, Reinforcement, Optimism, Emotion and Goals* domains were notably absent. Meanwhile, domains such as *Skills, Beliefs about Capabilities, Beliefs about Consequences, Memory, Attention and Decision Processes, Environmental Context and Resources* and *Behavioural Regulation* were fairly represented across the identified strategies.

## Discussion

With the increasing integration of genetic practices in healthcare, it is important that healthcare professionals are ready to engage with genetics to ensure patients have access to improved healthcare through the early identification of genetic conditions and provision of personalised care. This systematic review synthesised findings from 28 studies that explored both empirical and conceptual strategies supporting genetic practices among healthcare professionals and mapped these identified strategies onto the TDF. Educational strategies were the most commonly reported, while the most represented TDF domains were *Knowledge*, *Social Influences*, and *Social/Professional Role and Identity*.

Most studies reported the use of workshops, training programs, continuing education, and online learning. This aligns with past literature identifying a lack of knowledge and competence as key barriers to genetic integration across clinical roles [18, 24]. The studies included in the review indicated the effectiveness of these educational strategies [43, 44, 52]. For instance, Kronk et al. showed that a training program significantly increased genomic competencies [33]. However, it is important to note that studies employed diverse educational methods varied in content and delivery format [43, 44, 52].

The conceptual strategies suggested approaches related to raising awareness and fostering attitudes [24, 40, 47]. For instance, the *Belief about Consequences* domain was reflected through the need to see the value in genetic diagnoses to better support clinical decision-making among speech pathologists [24]. These findings suggest that it is essential for healthcare professionals to understand the potential impact of genetics. In turn, they may be more inclined to adopt genetic practices. Raising awareness of the clinical relevance and long-term benefits of genetics could play an important role in shifting attitudes towards incorporating genetics into clinical practice among healthcare professionals.

Interestingly, conceptual strategies were more commonly reported than empirical strategies in this review, suggesting there is a lack of evaluation and evidence of effectiveness among the identified strategies. TDF/ domains such as *Emotion, Goals, Reinforcement, optimism* and *Intentions*, were absent from the strategies identified, suggesting these domains have been overlooked. Affective responses, such as the *Emotion* domain, entail emotional reactions such as fear, anxiety, and discomfort [27], which can decrease healthcare professionals’ willingness to engage in genetic discussion with patients even when they are equipped with genetic *Knowledge* and *Skills*. Similarly, the lack of *Reinforcement, Intention, Optimism*, and *Goals* may reduce motivation to continue engaging in genetic practices [27]. These underrepresented domains may reflect broader challenges within integrating genetics. The absence of these domains suggests that current research may be disproportionately focused on knowledge-based interventions perhaps because they are easier to implement and measure, whereas affective and motivational determinants are less frequently examined despite their importance for sustained behaviour change.

### Limitations

Our systematic review contributes to the literature on identifying strategies to support the integration of genetic practices among healthcare professionals. However, there are several limitations that should be considered. While we aimed to capture a broad range of strategies, the inclusion of peer-reviewed literature published from 2020 onwards may have excluded relevant earlier studies that could have provided additional insights. Most of the included studies focused on nurses, with fewer studies involving healthcare professionals such as speech pathologists, occupational therapists, or optometrists. As a result, the findings may be more reflective of the nursing context than that of other professional groups.

By mapping strategies onto the TDF domains, we were able to recognise which aspects of behaviour have been targeted to date to support healthcare professionals, and which are missing. Future research should explore how domains such as *Beliefs about Consequences, Intentions, Goals, Emotion*, and *Reinforcement* support the integration of genetic practices in real-world settings. Future studies could evaluate these domains more explicitly by assessing allied health care professionals’ *Intentions* (e.g. stated plans or likelihood of incorporating genomics into routine care), *Reinforcement* (e.g. the presence of feedback, incentives, or recognition that supports continued use of genomics), *Optimism* (e.g. confidence in their ability to correctly interpret genomic results or beliefs about the value of genomics in improving patient outcomes), *Goals* (e.g. the extent to which clinicians prioritise genomics among competing clinical responsibilities), and *Emotion* (e.g. measuring anxiety or discomfort associated with discussing genetic risk or delivering genomic test results). Incorporating these behavioural indicators would provide a more comprehensive understanding of the factors influencing engagement in genetic practices. There is also a need to explore and test the various conceptual strategies proposed in practice among nurses and other allied health professionals. In addition, it is necessary to scale up successful empirical strategies in real-world settings to strengthen the evidence-based intervention.

Research must address barriers such as time constraints, resource constraints and competing clinical demands. Strengthening collaboration between researchers, clinicians, consumers, and policy stakeholders will also be essential to ensure that future strategies are feasible and relevant for the integration of genetics.

### Summary

The findings of this review highlight a need for greater diversity in the implementation strategies used to support genetic integration among health care professionals. While improving genetic literacy is important, our findings highlight the need for strategies to address affective, social, and environmental determinants. The review also suggests that healthcare professionals may benefit from support that is beyond *Knowledge* and *Social Influences* as including strategies that acknowledge emotional responses, strengthen motivation and intentions, can improve their genetic literacy and support the integration of genetics into practice. TDF mapping highlights gaps in the current interventions that support genetic practices among allied healthcare professionals and nurses. The review offers a more targeted insight into guidelines for the development of future interventions to ensure strategies more comprehensively target the determinants influencing genetic practice.

## Supplementary information

Supplementary file 1 Detailed Search Strategy

Supplementary file 2 Trial Strategies
