# Supplementary material for: Promoting genetic and genomic practices among allied healthcare professionals and nurses: a systematic review
Source: Eur J Hum Genet. 2026 Feb 27;34(5):583–96. doi: 10.1038/s41431-026-02038-5 (PMC13171888; doi:10.1038/s41431-026-02038-5)
Supplement: Supplementary file 1 — Detailed Search Strategy [file 41431_2026_2038_MOESM1_ESM.docx]

**Supplementary file 1. Detailed Search Strategy.**

Key search terms were guided by the review question and inclusion criteria. The search strategy involved developing strings of terms and synonyms. A comprehensive and systematic literature search was conducted in six electronic databases: CINAHL (EBSCOhost), MEDLINE (Ovid), Embase (Ovid), Emcare (Ovid), Scopus, and Web of Science.

**PICO- Formulate search strategy**

**Population-** nurses, allied healthcare professionals, speech pathologists
occupational therapists, audiologists, physiotherapists, optometrists **Intervention -** Strategies to support integration in genetic or genomic practices.
**Comparison -** no intervention
**Outcome -** Engagement in genetic or genomic practices/ strategies to implement in practices.

**Table 1** Search strategy for all databases.

| **Database** | **Platform** | **Search Strategy** | **Results** |
| --- | --- | --- | --- |
| CINAHL | EBSCOhost | (genomic* OR genetic) W2 (referral OR counsel* OR test* OR consult* OR practice* OR knowledge OR training OR education) AND ("speech pathologist*" OR "speech therapist*" OR "speech-language pathologist*" OR nurse* OR "nurse practitioner*" OR "clinical nurse*" OR "allied health professional*" OR "healthcare professional*" OR "health professional*" OR "allied healthcare provider*" OR "occupational therap*" OR audiolog* OR physiotherap* OR optometr*) AND (implementation OR policy OR policies OR guideline* OR strateg* OR role OR roles OR referral OR intervention* OR training OR workshop* OR education) | **871** |
| MEDLINE | Ovid | (genomic* OR genetic) adj2 (referral OR counsel* OR test* OR consult* OR practice* OR knowledge OR training OR education) AND (nurse* OR "nurse practitioner*" OR "speech pathologist*" OR "speech therapist*" OR "speech-language pathologist*" OR "allied health professional*" OR "healthcare professional*" OR "health professional*" OR "occupational therap*" OR audiolog* OR physiotherap* OR optometr*) AND (implementation OR policy OR policies OR guideline* OR strateg* OR role OR roles OR referral OR intervention* OR training OR workshop* OR education) | **1,824** |
| Embase | Ovid | (genomic* OR genetic) adj2 (referral OR counsel* OR test* OR consult* OR practice* OR knowledge OR training OR education) AND (nurse* OR "nurse practitioner*" OR "speech pathologist*" OR "speech therapist*" OR "speech-language pathologist*" OR "allied health professional*" OR "healthcare professional*" OR "health professional*" OR "occupational therap*" OR audiolog* OR physiotherap* OR optometr*) AND (implementation OR policy OR policies OR guideline* OR strateg* OR role OR roles OR referral OR intervention* OR training OR workshop* OR education) | **2,623** |
| Emcare | Ovid | (genomic* OR genetic) adj2 (referral OR counsel* OR test* OR consult* OR practice* OR knowledge OR training OR education) AND (nurse* OR "speech pathologist*" OR "speech therapist*" OR "speech-language pathologist*" OR "allied health professional*" OR "healthcare professional*" OR "health professional*" OR "occupational therap*" OR audiolog* OR physiotherap* OR optometr*) AND (implementation OR policy OR policies OR guideline* OR strateg* OR role OR roles OR referral OR intervention* OR training OR workshop* OR education) | **280** |
| Scopus | Elsevier | TITLE-ABS-KEY ( (genomic* OR genetic) W/2 (referral OR counsel* OR test* OR consult* OR practice* OR knowledge OR training OR education) AND ("speech pathologist*" OR "speech therapist*" OR "speech-language pathologist*" OR nurse* OR "nurse practitioner*" OR "allied health professional*" OR "healthcare professional*" OR "health professional*" OR "occupational therap*" OR audiolog* OR physiotherap* OR optometr*) AND (implementation OR policy OR policies OR guideline* OR strateg* OR role OR roles OR referral OR intervention* OR training OR workshop* OR education) ) | **1,194** |
| Web of Science | Clarivate | TS( (genomic* OR genetic) NEAR/2 (referral OR counsel* OR test* OR consult* OR practice* OR knowledge OR training OR education) AND ("speech pathologist*" OR "speech therapist*" OR "speech-language pathologist*" OR nurse* OR "nurse practitioner*" OR "allied health professional*" OR "healthcare professional*" OR "health professional*" OR "occupational therap*" OR audiolog* OR physiotherap* OR optometr*) AND (implementation OR policy OR policies OR guideline* OR strateg* OR role OR roles OR referral OR intervention* OR training OR workshop* OR education) ) | **1,723** |
