# Supplementary material for: Promoting genetic and genomic practices among allied healthcare professionals and nurses: a systematic review
Source: Eur J Hum Genet. 2026 Feb 27;34(5):583–96. doi: 10.1038/s41431-026-02038-5 (PMC13171888; doi:10.1038/s41431-026-02038-5)
Supplement: Supplementary file 2 — Trial Strategies [file 41431_2026_2038_MOESM2_ESM.docx]

| **Category** | **Preferred Strategies** | **Quote** |
| --- | --- | --- |
| Structured learning | Self-directed interactive learning (n= 7) | “I do find that self-directed interactive learning is… is very doable and can fit in with my workload and with my professional development plan. So, particularly if it's structured in that more, like, you know, on-demand, like, module-based. Sort of online interactive learning, I find that really, really helpful as well.”  *P8* “...I love that I can log on and do an hour, or… wake up early and do an hour here. It just means that I actually saw as something feasible for me to do, rather than having to get time off” *P3* |
| Resource format | Flowcharts (n= 6) | “I love a flow chart. Step by step clinical guidelines and yeah, and flow charts I think for me” *P10*  “…Either step by step guidelines or flow charts I think would be the most useful.” *P1* |
|  | Step by step guidelines (n = 5) |  |
| Delivery mode | Synchronous learning (n =7) | “I guess synchronous is…. Yeah, ideal. Um, you can ask questions, you can talk through things, you…. Learn through interacting and…. Having conversations with others. Um, certainly it is less convenient, but…. You learn more.” *P7*  “..To be honest, I most prefer learning face-to-face. Synchronously, but I think the one that is most realistic for people who might be working in lots of different professions Uh, of different clinical spaces, and then also making it as equitable as possible. So that even if you're regional or remote, you could access the training.” *P3* |
| Learning activities | Shadow/observe clinician (n = 8) | “I personally learn probably best either shadowing, observing a clinician or group discussions, that's what I like” *P9*  “I think group discussion is also really helpful as well, because generally, as clinicians, we can sort of build on others' ideas and thoughts…” *P8* |
|  | Group discussion (n= 7) |  |
| Practical tools | Location, proximity, and access to other clinicians (n =10) | “..access to almost like other clinicians with their genetic counsellors. I think that really makes a difference as speech pathologists, and it's an era that's very much lacking...” *P4*  “..I think yeah, location and proximity to other clinicians is probably what drives most of my referrals that currently...” *P5* |
